# Overlap between dengue, Zika and chikungunya hotspots in the city of Rio de Janeiro

**DOI:** 10.1371/journal.pone.0273980

**Published:** 2022-09-06

**Authors:** Eny Regina da Silva Queiroz, Roberto de Andrade Medronho

**Affiliations:** 1 Instituto de Estudos em Saúde Coletiva, Universidade Federal do Rio de Janeiro, Rio de Janeiro, Rio de Janeiro, Brazil; 2 Faculdade de Medicina, Universidade Federal do Rio de Janeiro, Rio de Janeiro, RJ, Brazil; Fundacao Oswaldo Cruz, BRAZIL

## Abstract

**Background:**

Arboviruses represent a threat to global public health. In the Americas, the dengue fever is endemic. This situation worsens with the introduction of emerging, Zika fever and chikungunya fever, causing epidemics in several countries within the last decade. Hotspot analysis contributes to understanding the spatial and temporal dynamics in the context of co-circulation of these three arboviral diseases, which have the same vector: *Aedes aegypti*.

**Objective:**

To analyze the spatial distribution and agreement between the hotspots of the historical series of reported dengue cases from 2000 to 2014 and the Zika, chikungunya and dengue cases hotspots from 2015 to 2019 in the city of Rio de Janeiro.

**Methods:**

To identify hotspots, Gi* statistics were calculated for the annual incidence rates of reported cases of dengue, Zika, and chikungunya by neighborhood. Kendall’s W statistic was used to analyze the agreement between diseases hotspots.

**Results:**

There was no agreement between the hotspots of the dengue fever historical series (2000–2014) and those of the emerging Zika fever and chikungunya fever (2015–2019). However, there was agreement between hotspots of the three arboviral diseases between 2015 and 2019.

**Conclusion:**

The results of this study show the existence of persistent hotspots that need to be prioritized in public policies for the prevention and control of these diseases. The techniques used with data from epidemiological surveillance services can help in better understanding of the dynamics of these diseases wherever they circulate in the world.

## 1. Introduction

Arboviruses represent a threat to public health across the world. In the Americas, the dengue fever circulates endemically, simultaneously with the newly emerged diseases, Zika fever and chikungunya fever, with epidemics recorded in several countries in the last decade [1, 2]. Other arboviral diseases such as West Nile fever, yellow fever, and Mayaro fever, have also been recorded and have the potential to spread to urban centers causing explosive epidemics [1].

Among the main vectors of diseases relevant to public health, the *Aedes aegypti* and *Aedes albopictus* mosquitoes stand out. They are capable of transmitting the virus of dengue, Zika, chikungunya, yellow fever, and others. Present on all continents, there are still several locations with favorable conditions for the introduction of these species, which have expanded their geographic distribution around the world [3].

Understanding the dynamics of arboviral diseases in the context of co-circulation represents a challenge as it involves host factors such as immunity, mobility and behavior, vectors, viruses, and the environment. Starting from the premise of the heterogeneous distribution of arboviral diseases and their determining factors, several studies have been carried out with the objective of investigating spatial, temporal, or spatiotemporal patterns of these diseases [4–12].

Methods that seek to detect spatial clusters are able to identify areas of greater risk and increase understanding of the dynamics of certain phenomena. This approach has already been used for investigation of spatial and temporal patterns of dengue fever and its underlying factors [4, 6], prospective analysis, monitoring and detection of epidemic processes in real time [7], and detection and overlapping of hotspots of dengue fever, Zika fever, and chikungunya fever [8–10].

In Mérida, Mexico, Bizanzio et al. [8] evaluated the spatiotemporal overlap between dengue, Zika, and chikungunya cases. They also investigated whether persistent hotspots with historical dengue data coincided with hotspots of the emerging diseases, Zika, and chikungunya. In a similar study, Dzul-Mazanlila et al. [9] analyzed hotspots of these three arboviral diseases in various cities in Mexico. Another study in Dominican Republic, evaluated the temporal pattern of dengue, Zika, and chikungunya epidemics between 2012 and 2018 [11]. In the city of Fortaleza, Kazazian et al. [12] evaluated the spatiotemporal dynamics of these three diseases between 2011 and 2017. Areas of persistent dengue risk in populations with different patterns of mobility and immunity have been investigated in the city of Rio de Janeiro, [4]. The overlap of dengue, Zika, and chikungunya clusters during the first occurrence of a triple epidemic have also been analyzed [10].

With the lack of vaccines for most arboviral diseases [13], with the exception of yellow fever, the main public health strategies related to these diseases involve vector control and the organization of health care system for diagnosis and management of suspected cases.

Due to the complexity of the factors involved in the transmission and spread of arboviral diseases, the spatial and temporal dynamics in the context of co-circulation need to be better understood in order to support actions for the prevention of these epidemics.

We aimed to describe and analyze the spatial distribution and overlap of hotspots in the historical series of reported cases of dengue fever from 2000 to 2014 with reported cases of Zika fever and chikungunya fever in the period from 2015 to 2019 in the city of Rio de Janeiro. In addition to this first objective, we aimed to describe and analyze the spatial distribution and overlap of hotspots in the context of the co-circulation of the three diseases in the period from 2015 to 2019 in the city of Rio de Janeiro.

### 2. Materials and methods

The city of Rio de Janeiro is located at 22°54’23" south latitude and 43°10’21" west longitude. It is the second most populated city in the country and the most populous in the state of Rio de Janeiro, with an estimated population of 6,747,815 in 2020 [14]

With many tourist attractions, the city is home to the only international airport in the state and receives visitors from other states and countries. It is characterized by an intense population movement from other cities, using road and rail transport networks, in search of work and access to public and private services.

**Fig 1:**
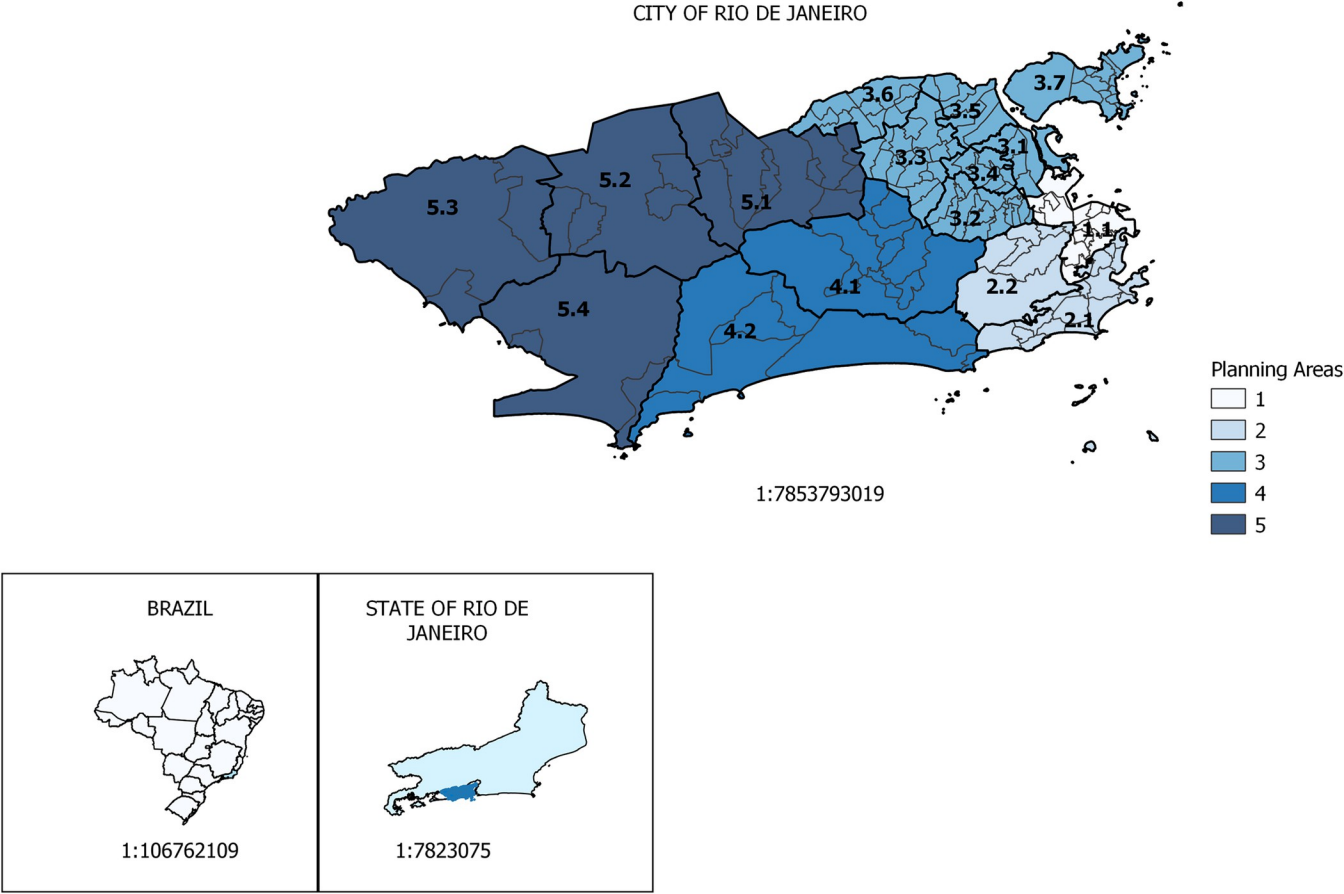
Map of the city of Rio de Janeiro. Planning areas and their subdivisions.

The territory of the city (Fig 1) is administratively divided into 160 neighborhoods, distributed into five planning areas (AP), according to environmental, historical, geographic and land use, and occupation characteristics. The APs are subdivided, according to criteria of homogeneity, into sixteen planning regions [15], designated as AP.

The city of Rio de Janeiro is marked by social, demographic, and environmental heterogeneities (S1 Fig). The city has a high municipal human development index (MHDI) (0.799). The social development index (SDI) is a composite indicator, similar to the MHDI, that collates data on income, sanitation, and schooling, was equal to 0.609 in 2021. This index ranges from 0 to 1, with 0 being the worst situation and 1 being the best situation. AP 2.1 had the highest SDI (0.722), while AP 5.4 had an SDI below 0.50. These data are available on the digital platform of the municipal government of Rio de Janeiro—DATA.RIO [15]. For additional information regarding this, see S1 Table in the Supporting information.

### 2.1. Data source

All cases of dengue between 2000 and 2019 and of Zika and chikungunya between 2015 and 2019 that were reported to the Information System on Diseases of Compulsory Declaration (SINAN) were included in the study. The data is aggregated by neighborhoods and by AP and is available on the website of the Municipal Health Department (http://www.rio.rj.gov.br/web/sms). The dengue, Zika, and chikungunya are notifiable diseases in Brazil. All suspected cases of these diseases, as defined by the Brazilian Ministry of Health [16], must be registered with SINAN to be investigated for infection, according to laboratory or clinical-epidemiological criteria. Each case is either confirmed or, if suspicions did not prove to be true, discarded.

Despite having mostly symptomatic cases, often without laboratory confirmation, data from SINAN have been used in several studies [10, 17–19] to describe and analyze the dynamics of infectious diseases in populations. Although the data have limitations, they are still representative of the trend of these diseases [10]. The digital cartographic grids and data related to population, slums boundaries, land use, characterization of neighborhoods, and their subdivisions come from the Brazilian Institute of Geography and Statistics and extracted from the DATA.RIO platform. Annual incidence rates of dengue fever, Zika fever, and chikungunya fever, were calculated for the study period, considering the number of cases per 100,000 inhabitants.

### 2.2. Analyses

#### 2.2.1. Identification of hotspots

Gi* statistics were calculated for the annual incidence rates of reported cases of dengue, Zika, and chikungunya by neighborhoods. In each year, the neighborhoods that presented Gi* with statistical significance were categorized as hotspots. This procedure was performed using the GEODA software.

Getis and Ord created local statistics Gi and Gi* that *allow for the detection of pockets of spatial association that may not be evident when using global statistics* [20]. The Gi* statistic for each area is defined by the equation:

$$Gi^{*}=\frac{\Sigma_{jW_{ij}y_{j}}}{\Sigma_{j}\;y_{j}}$$ (1)

where *w*_*ij*_ is the weighting matrix defined by distance or other neighborhood criteria, and *y*_*j*_ is the value of the random variable in each spatial unit. The difference between Gi and Gi* is that Gi* includes the value of the study variable observed in the reference unit in the numerator.

The interpretation of this statistic is given through the standardized variable Z(Gi*):

$$Z(Gi^{*})=\frac{Gi^{*}-E(Gi^{*})}{DP(Gi^{*})}$$ (2)

where E(Gi*) is the expected value of Gi* and DP(Gi*) is its standard deviation. Positive and significant values of Gi* indicate areas with high values for a given variable surrounded by neighbors with high values (hotspots). Negative and significant values of Gi* indicate areas with low values surrounded by neighbors with low values (coldspots) [20]. Thematic maps were constructed indicating the classification of neighborhoods, according to the number of hotspots and coldspots per period. These procedures were performed using QGIS 3.10.4 software.

#### 2.2.2. Agreement analyses

Kendall’s W statistic assesses the agreement among ordinal variables and can range from 0 (no agreement) to 1 (perfect agreement) [21]. Kendall’s W statistic was calculated for the following analyses:

- Analyzing the overlap between the hotspots of the dengue fever historical series (2000 to 2014) with the Zika fever and chikungunya fever hotspots (2015 to 2019)
- Analyzing the overlap between the hotspots of the dengue fever historical series (2000 to 2014), considering only the epidemic years in this period, with the Zika fever and chikungunya fever hotspots (2015 to 2019)
- Analyzing the overlap between dengue, Zika, and chikungunya diseases hotspots during the co-circulation period (2015 to 2019)

Analyses were performed using the ‘kendall’ command (correct option) from the irr package of the R software, version 4.0.5.

## 3. Results

**Fig 2:**
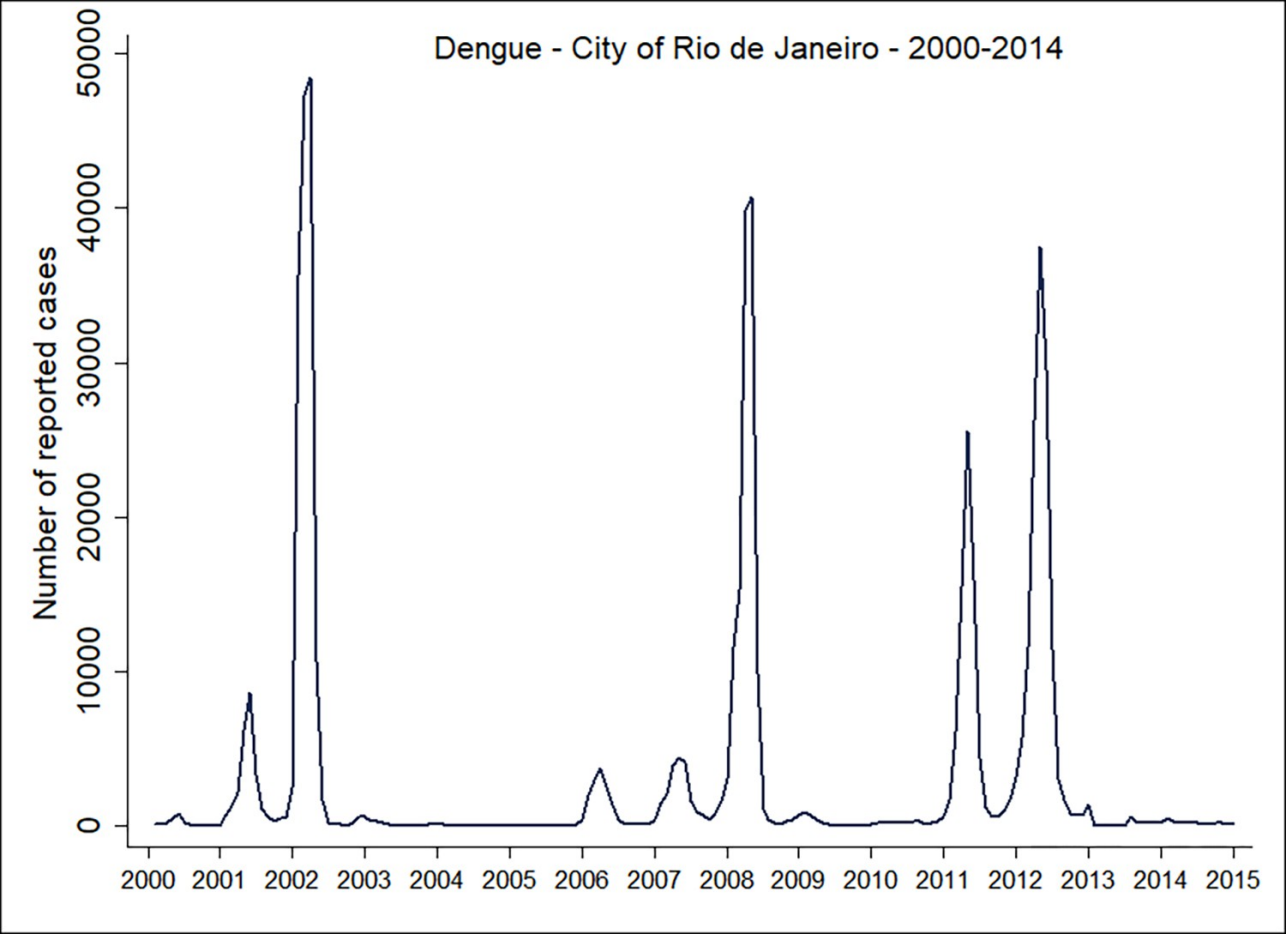
Epidemic curve of reported cases of dengue fever in the city of Rio de Janeiro by date of onset of symptoms 2000–2014.

**Fig 3:**
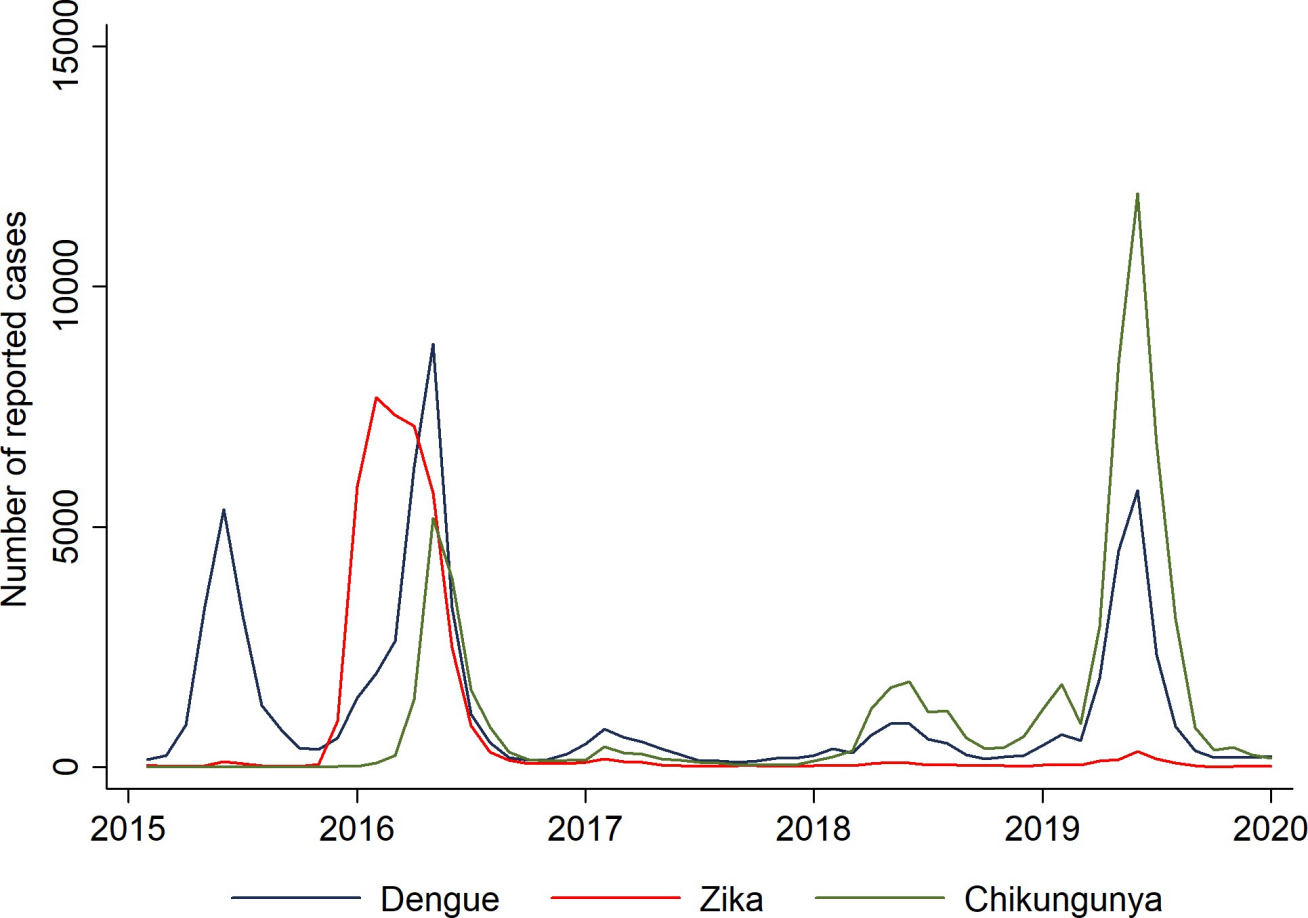
Epidemics curves of reported cases of dengue fever, Zika fever, and chikungunya fever, by date of onset symptoms in the city of Rio de Janeiro. 2015–2019.

In the city of Rio de Janeiro, between 2000 and 2014, the highest peaks of dengue fever notifications were observed in 2002, 2008, 2011, and 2012, which corresponded to the epidemic years (Fig 2). In 2015, there was an increase in dengue cases, and at the end of this year an increase in Zika cases with an epidemic peak in 2016. This also coincided with the increase in dengue and chikungunya cases. After 2016, Zika transmission reduced and remained low. In 2019, there was a new increase in chikungunya and dengue cases (Fig 3). See S2 Table for more information.

**Fig 4:**
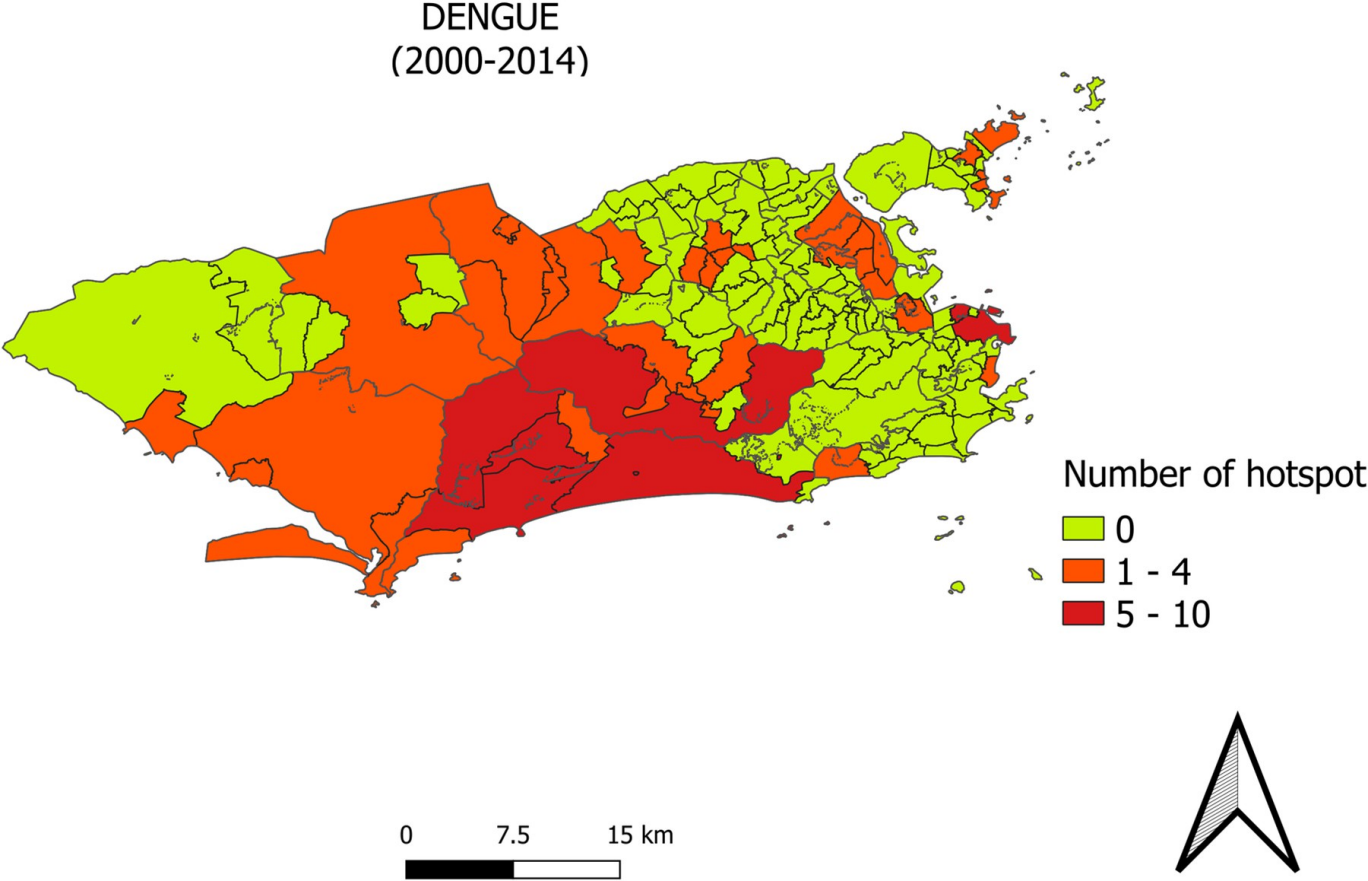
Hotspots of the annual incidence rates of reported cases of dengue fever by neighborhood in the city of Rio de Janeiro from 2000 to 2014.

Fig 4 shows the presence of dengue incidence rate hotspots in the period between 2000 and 2014 in the following programmatic areas: AP3 (3.1—Ramos, 3.3—Madureira and 3.7 –Ilha do Governador); AP5 (5.1—Bangu, 5.2—Campo Grande and 5.3—Santa Cruz); AP4 (4.1—Jacarepaguá, 4.2—Barra da Tijuca); and AP1 (Centro). The S2 Fig shows the spatial distribution of annual dengue fever hotspots in this period.

**Fig 5:**
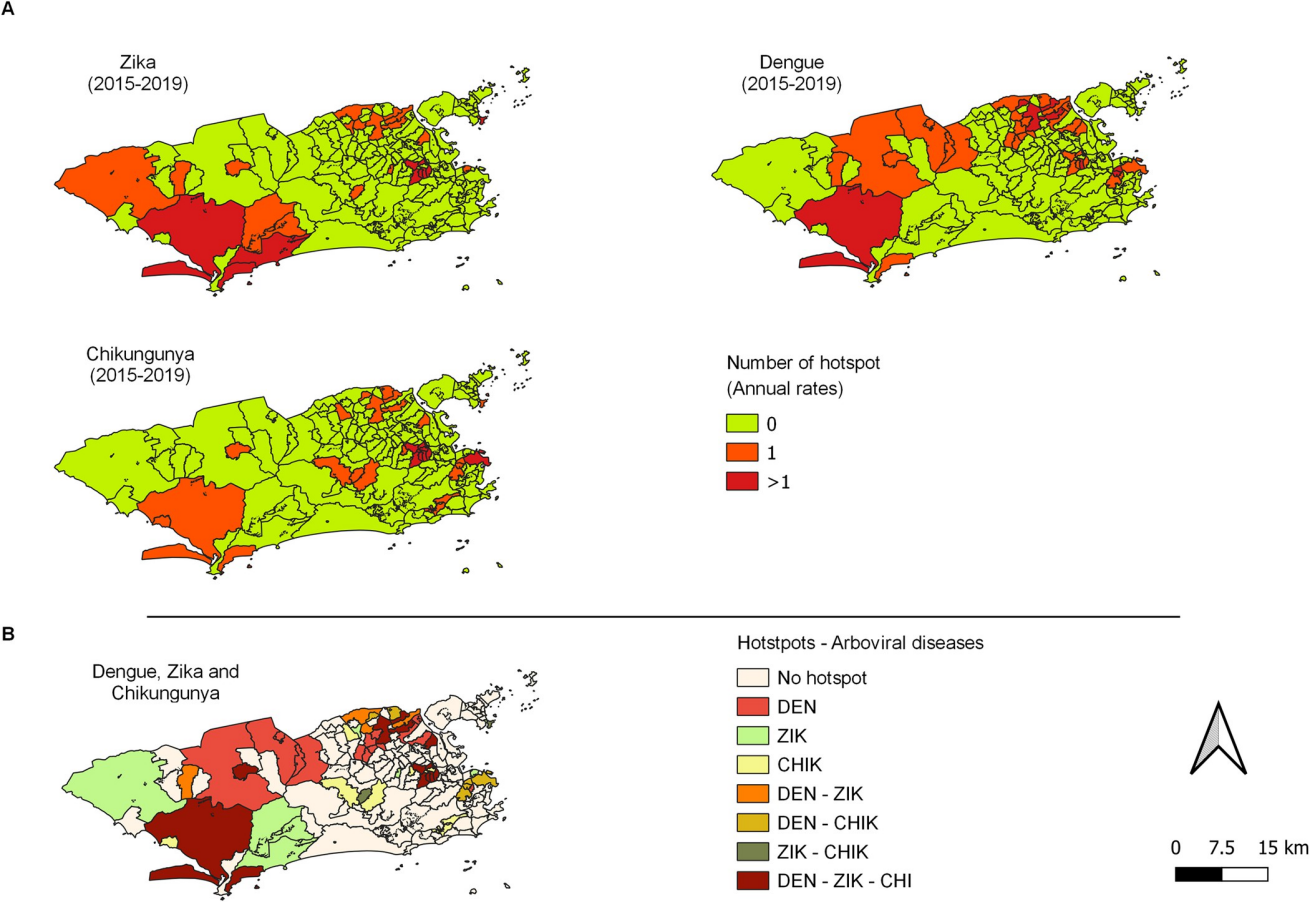
Hotspots of the annual incidence rates of reported cases of dengue fever, Zika fever, and chikungunya fever, by neighborhood in the city of Rio de Janeiro from 2015 to 2019. A–Number of hotspots. B–Hotspots observed by disease and co-circulation.

Between 2015 and 2019, the presence of dengue, Zika, or chikungunya hotspots was observed predominantly in neighborhoods located in APs 3 and 5. The neighborhoods that had the highest number (3 or 4) of hotspots in this period by disease were: dengue—Guaratiba, (AP 5); chikungunya—Rocha, Jacarezinho, Riachuelo, and Sampaio (AP3); and Zika–Guaratiba (AP 5), Grumari (AP4) and Ribeira (AP3) (Fig 5 and S3 Fig).

Table 1 presents Kendall’s W statistic for the hotspot count of dengue incidence rates from 2000 to 2014, as well as for only the dengue epidemic years in the same period. Also presented in Table 1 is Kendall’s W statistic for the hotspot count of dengue, Zika, and chikungunya incidence rates by neighborhood in the city of Rio de Janeiro from 2015 to 2019. There was no significant agreement between the dengue hotspots (historical series from 2000 to 2014) and the Zika and chikungunya hotspots from 2015 to 2019. However, there was agreement with borderline significance between dengue hotspots from 2000 to 2014 and dengue hotspots between 2015 and 2019 (p = 0.09). There was agreement between the dengue hotspots in the epidemic years from 2000 to 2014 with the Zika hotspots in the period from 2015 to 2019 (p = 0.09). There was also a significant agreement between dengue hotspots form the epidemic years in 2000–2014 and the dengue hotspots from 2015–2019 (p = 0.04). There was significant agreement between the hotspots of the incidence rates of dengue, Zika, and chikungunya in the context of co-circulation of the three arboviral diseases in the period from 2015 to 2019 (p<0.001; Table 1).

**Table 1:** Kendall’s W statistics for the hotspot count of dengue incidence rates from 2000 to 2014; dengue epidemic years for the same period; and dengue, Zika, and chikungunya incidence rates by neighborhood in the city of Rio de Janeiro from 2015 to 2019.

		Zika	Chikungunya	Dengue
		(2015–2019)	(2015–2019)	(2015–2019)
Hotspots	Dengue	0.53	0.51	**0.577**
	(2000–2014)	p = 0.29	p = 0.47	p = 0.09
	Dengue (epidemic years)	**0.58**	0.5	**0.6**
	(2000–2014)	**p = 0.09**	p = 0.30	p = 0.04
	Dengue	**0.72**	**0.75**	
	(2015–2019)	p = 0.00	p = 0.00	
	Chikungunya	**0.74**		
	(2015–2019)	p = 0.00		

## 4. Discussion

In this study, using data provided by epidemiological surveillance services and a set of spatial analysis techniques, it was possible to identify agreement between the location of hotspots of the incidence rates of the three diseases (dengue, Zika, and chikungunya) in the period from 2015 to 2019. No agreement was found between the hotspots of the annual incidence rates of the dengue historical series (2000 to 2014) with the incidence rates of Zika and chikungunya from 2015 to 2019.

Some authors also found differences between spatial patterns of a historical series of dengue and other emerging arboviral diseases. Kazazian et al [12] found differences in the spatial pattern of chikungunya with a previous pattern of dengue. Another study carried out in the Dominican Republic observed spatiotemporal asynchronicity between the three diseases and concluded that the spatial pattern of dengue cannot be used to predict the location of emerging arboviral diseases [11]. The authors suggested that in the emergence of a new arbovirus, population susceptibility to the virus exerts a greater influence on the occurrence of cases than other factors associated with the vector, such as meteorological factors. In the city of Rio de Janeiro, when the co-circulation of dengue, Zika, and chikungunya diseases was recorded for the first time, it was believed that the entire population would be susceptible to the chikungunya and Zika arboviruses; however, several locations in the city had immunity to dengue due to the previous circulation of the four serotypes of the virus. Yet Bizanzio et al [8] found a different result in the city of Mérida in Mexico. These authors observed that the first cases of emerging diseases Zika and chikungunya were recorded in census tracts where dengue hotspots were observed in previous years.

From 2000 to 2011, in the years considered an epidemic, different serotypes of the dengue virus predominated—2002: DENV-3, 2008: DENV-2, 2011: DENV-1 [4]. However, the annual dengue hotspots in this period were mainly concentrated in AP5 and AP4. In 2012, with the introduction of the DENV-4 serotype, there was a predominance of hotspots in AP3 neighborhoods. A study pointed out that during epidemics with a predominance of one serotype in human cases, the other serotypes remain circulating in the vector population [22]. In this way, the accumulation of people susceptible to a particular dengue virus serotype in a locality can favor an increase in cases of the disease [4] and the occurrence of new epidemics.

Regarding the analysis of the overlap from the first record of the triple epidemic from 2015 to 2019, our study found a similar result with two other studies carried out in cities in Mexico, in which overlap of the clusters of the three arboviral diseases was observed [8, 9]. Other authors, however, did not find any overlap between the diseases [10–12]. This indicates that during co-circulation, each arboviral disease would possibly predominate in distinct regions and that, even if there is an overlap of hotspots in space on an annual scale, these clusters would have occurred in different months [12]. Although, on a finer temporal scale, these disease clusters occurred in different months, our analysis suggests the existence of areas with persistent presence of the vector, predominantly in AP3 and AP5 districts of the city of Rio de Janeiro.

One study found a predominance of dengue, Zika, and chikungunya clusters in areas of high population density and low socioeconomic status in the municipality of Rio de Janeiro [10]. Another study found a negative association between vegetation cover and dengue incidence rates in Bhutan [23]. In the present study, the clusters observed in AP3 are located in areas with low vegetation cover and high demographic densities. However, clusters were also observed in neighborhoods with low demographic densities in AP5, coinciding with another study on dengue in Rio de Janeiro. Therefore, the authors considered that the high risk for dengue in AP5 could be due to a recent and accelerated urban growth without adequate infrastructure [4].

In this study, data from passive epidemiological surveillance were used, which excludes subclinical cases that did not seek medical attention, or cases that were not reported. In epidemic periods, to optimize resources, only a sample of symptomatic cases are confirmed in the laboratory. The aim of this is to identify circulating serotypes in the case of dengue, as well as to enable the differential diagnosis between the three diseases in the case of their co-circulation. The similarity between the symptoms of the three arboviral diseases and the absence of laboratory confirmation leads to misclassification bias. This fact reinforces the need to improve the ability of identifying and diagnosing arboviral diseases [19, 24].

Despite these limitations, it was possible to identify areas with persistent circulation of arboviral diseases. These areas need to be prioritized by managers, considering that dengue, Zika, and chikungunya circulate endemically, in addition to the risk of introducing other arboviral diseases, such as yellow fever. Techniques used are accessible and can be used by local epidemiological surveillance services to support the planning of effective actions to control the vector and the disease.

## Supporting information

S1 Fig Thematic maps of the city of Rio de Janeiro. A–Demographic density by neighborhoods (quintiles); B—Land use; C- Slums boundaries. 2010.
(TIF)

S2 Fig Hotspots and coldspots of dengue fever. City of Rio de Janeiro. 2000–2014.
(TIF)

S3 Fig Hotspots and coldspots of dengue fever, Zika fever and chikungunya fever. City of Rio de Janeiro. 2015–2019.
(TIF)

S1 Table The social development index (SDI) and per capita income in minimum wages by planning areas. City of Rio de Janeiro. 2010.
(DOCX)

S2 Table Summary of the annual incidence rates of reported cases of dengue fever, Zika fever, and chikungunya fever, by neighborhoods. City of Rio de Janeiro. 2015–2019.
(DOCX)
