# Supplementary figures and images for: Overlap between dengue, Zika and chikungunya hotspots in the city of Rio de Janeiro
Source: PLoS One. 2022 Sep 6;17(9):e0273980. doi: 10.1371/journal.pone.0273980 (PMC9447914; doi:10.1371/journal.pone.0273980)

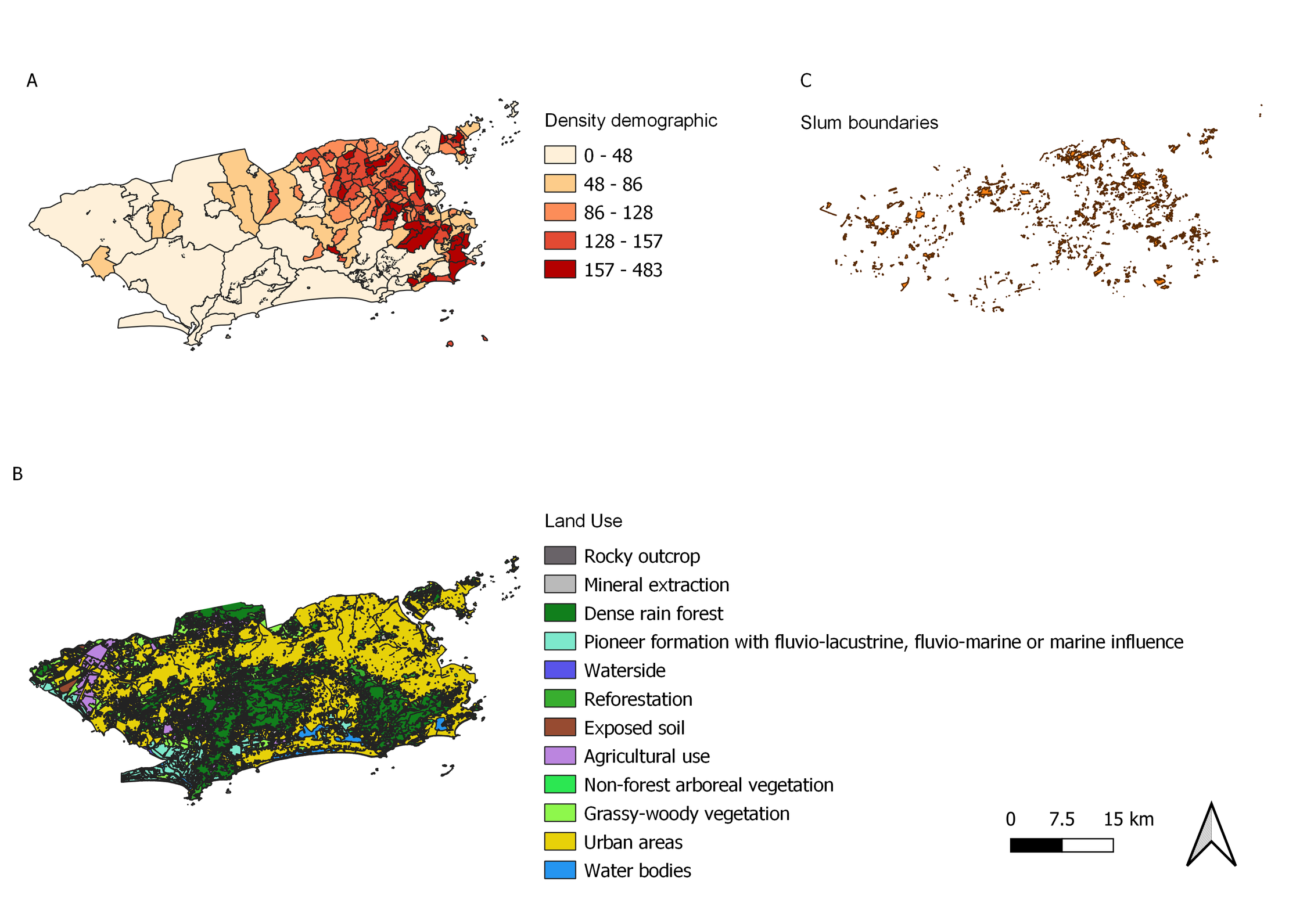

Supplement: S1 Fig — A–Demographic density by neighborhoods (quintiles); B—Land use; C- Slums boundaries. 2010. (TIF) [file pone.0273980.s001.tif]

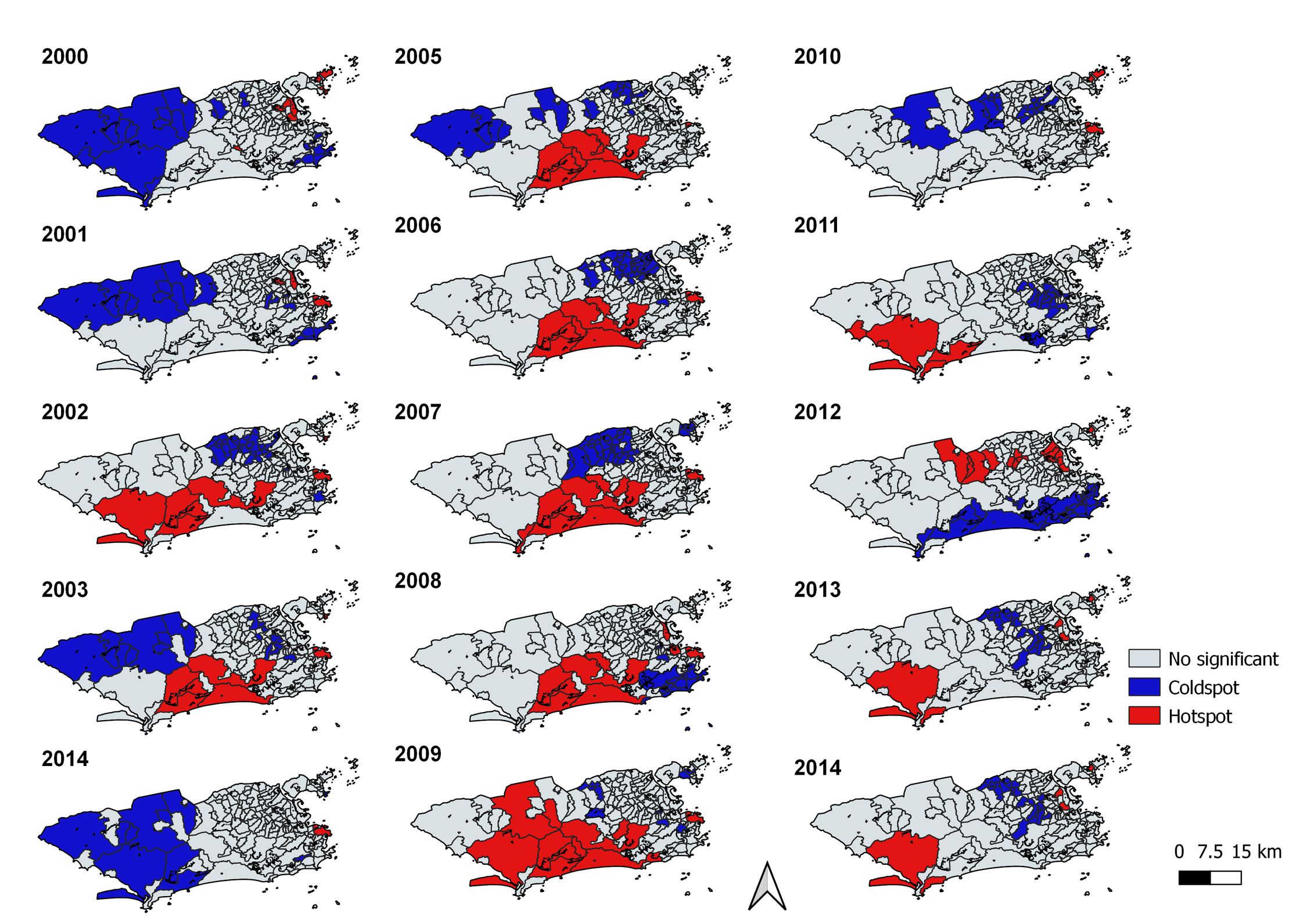

Supplement: S2 Fig — City of Rio de Janeiro. 2000–2014. (TIF) [file pone.0273980.s002.tif]

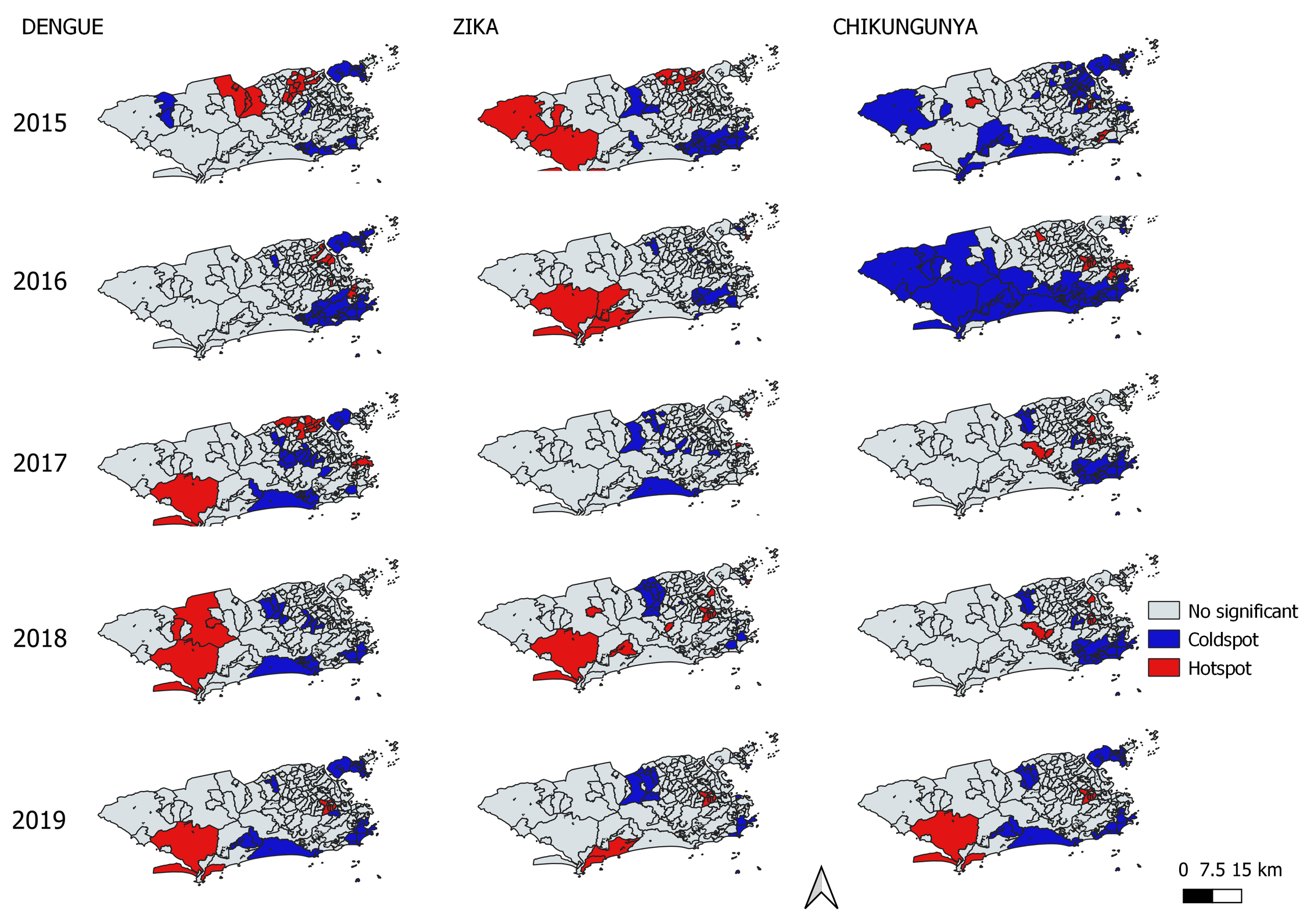

Supplement: S3 Fig — City of Rio de Janeiro. 2015–2019. (TIF) [file pone.0273980.s003.tif]
