# Supplementary material for: Overlap between dengue, Zika and chikungunya hotspots in the city of Rio de Janeiro
Source: PLoS One. 2022 Sep 6;17(9):e0273980. doi: 10.1371/journal.pone.0273980 (PMC9447914; doi:10.1371/journal.pone.0273980)
Supplement: S1 Table — City of Rio de Janeiro. 2010. (DOCX) [file pone.0273980.s004.docx]

S1 Table - The social development index (SDI) and per capita income in minimum wages by planning areas. City of Rio de Janeiro. 2010

| Planning areas | Social development index | Per capita income in minimum wages |
| --- | --- | --- |
| Município do Rio de Janeiro | 0.609 | 2270 |
| Planning area 1 | 0.600 | 1689 |
| Planning area 2 | 0.710 | 5280 |
| Planning area 3 | 0.591 | 1526 |
| Planning area 4 | 0.624 | 3201 |
| Planning area 5 | 0.554 | 1128 |
| Planning region 1.1 - Centro | 0.600 | 1689 |
| Planning region 2.1 - Zona Sul | 0.722 | 6033 |
| Planning region 2.2 - Tijuca | 0.688 | 3983 |
| Planning region 3.1 - Ramos | 0.571 | 1141 |
| Planning region 3.2 - Méier | 0.622 | 2115 |
| Planning region 3.3 - Madureira | 0.597 | 1575 |
| Planning region 3.4 - Inhaúma | 0.57 | 1182 |
| Planning region 3.5 - Penha | 0.577 | 1228 |
| Planning region 3.6 - Pavuna | 0.562 | 1060 |
| Planning region 3.7 - Ilha do Governador | 0.627 | 2291 |
| Planning region 4.1 - Jacarepaguá | 0.597 | 2008 |
| Planning region 4.2 - Barra da Tijuca | 0.676 | 5609 |
| Planning region 5.1 - Bangu | 0.572 | 1204 |
| Planning region 5.2 - Campo Grande | 0.562 | 1252 |
| Planning region 5.3 - Santa Cruz | 0.528 | 0.871 |
| Planning region 5.4 - Guaratiba | 0.493 | 0.945 |
