# Supplementary material for: Overlap between dengue, Zika and chikungunya hotspots in the city of Rio de Janeiro
Source: PLoS One. 2022 Sep 6;17(9):e0273980. doi: 10.1371/journal.pone.0273980 (PMC9447914; doi:10.1371/journal.pone.0273980)
Supplement: S2 Table — City of Rio de Janeiro. 2015–2019. (DOCX) [file pone.0273980.s005.docx]

S2 Table – Summary of the annual incidence rates of reported cases of dengue, Zika, and chikungunya by neighborhoods. City of Rio de Janeiro. 2015-2019

|  |  | Dengue | Zika | Chikungunya |
| --- | --- | --- | --- | --- |
| 2015 | Min | 0.0 | 0.0 | 0.0 |
|  | Q1 | 152.4 | 55.2 | 0.0 |
|  | Q2 | 220.0 | 95.6 | 0.0 |
|  | Q3 | 303.3 | 161.9 | 0 |
|  | Max | 1121.0 | 707.5 | 15.8 |
| 2016 | Min | 0.0 | 113.8 | 0.0 |
|  | Q1 | 169.9 | 322.9 | 86.9 |
|  | Q2 | 313.6 | 453.7 | 213.0 |
|  | Q3 | 491.0 | 632.0 | 355.1 |
|  | Max | 5232.6 | 4254.7 | 2302.7 |
| 2017 | Min | 0.0 | 0.0 | 0.0 |
|  | Q1 | 25.7 | 3.6 | 11.9 |
|  | Q2 | 47.5 | 8.8 | 21.4 |
|  | Q3 | 81.5 | 16.1 | 41.1 |
|  | Max | 254.0 | 119.0 | 267.8 |
| 2018 | Min | 0.0 | 0.0 | 0.0 |
|  | Q1 | 25.8 | 0.0 | 55.5 |
|  | Q2 | 50.9 | 6.4 | 104.0 |
|  | Q3 | 88.7 | 11.6 | 201.8 |
|  | Max | 1285.8 | 280.3 | 2792.1 |
| 2019 | Min | 0.0 | 0.0 | 0.0 |
|  | Q1 | 97.1 | 5.7 | 290.5 |
|  | Q2 | 179.7 | 15.1 | 482.7 |
|  | Q3 | 335.4 | 24.8 | 811.1 |
|  | Max | 7966.8 | 970.2 | 19954.7 |

Min – Minimum, Máx – Maximum, Q1 – Lower quartile, Q2 – Median, Q3 – Upper quartile.
